# Characterization and Electronic Properties of Heptazine Layers: Towards Promising Interfacial Materials for Organic Optoelectronics

**DOI:** 10.3390/ma13173826

**Published:** 2020-08-29

**Authors:** Issoufou Ibrahim Zamkoye, Houda El Gbouri, Remi Antony, Bernard Ratier, Johann Bouclé, Laurent Galmiche, Thierry Trigaud, Pierre Audebert

**Affiliations:** 1 University of Limoges, Centre National de la Recherche Scientifique, XLIM, UMR 7252, F-87000 Limoges, France; issoufou.ibrahim@etu.unilim.fr (I.I.Z.); houda.el-gbouri@etu.unilim.fr (H.E.G.); remi.antony@unilim.fr (R.A.); bernard.ratier@unilim.fr (B.R.); johann.boucle@unilim.fr (J.B.); 2 Laboratoire de Photophysique et Photochimie Supramoléculaires et Macromoléculaires UMR 5231, Centre National de la Recherche Scientifique, Ecole Normale Supérieure de Paris-Saclay, Rue de la Science, 91190 Gif s. Yvette, France; laurent.galmiche@ppsm.ens-cachan.fr

**Keywords:** heptazine, interfaces, KPFM, Fermi level, photoluminescence, organic solar cells

## Abstract

For the first time, an original compound belonging to the heptazine family has been deposited in the form of thin layers, both by thermal evaporation under vacuum and spin-coating techniques. In both cases, smooth and homogeneous layers have been obtained, and their properties evaluated for eventual applications in the field of organic electronics. The layers have been fully characterized by several concordant techniques, namely UV-visible spectroscopy, steady-state and transient fluorescence in the solid-state, as well as topographic and conductive atomic force microscopy (AFM) used in Kelvin probe force mode (KPFM). Consequently, the afferent energy levels, including Fermi level, have been determined, and show that these new heptazines are promising materials for tailoring the electronic properties of interfaces associated with printed electronic devices. A test experiment showing an improved electron transfer rate from a tris-(8-hydroxyquinoline) aluminum (Alq3) photo-active layer in presence of a heptazine interlayer is finally presented.

## 1. Introduction

Related to the problem of global warming, the implementation of renewable energy sources is currently a fundamental challenge to achieve sustainable development. Organic photovoltaic (OPV) is, from this point of view, recognized as one of the healthy solutions to meet the increase in energy needs. We can already put forward some properties such as mechanical flexibility, lightness, low cost, and low temperature manufacturing technique borrowed from printing industries [1,2]. Power conversion efficiency of over 17% [3] has already been achieved in single junction organic solar cells [4,5], thus legitimating hopes for a bright future.

A typical organic solar cell is formed by a multilayer stacking where a bulk heterojunction (BHJ) active layer is surrounded with two interfacial layers: a hole transport layer (HTL) and an electron transport layer (ETL). To date, the development of highly efficient OPV is mainly ensured by the synthesis and use of materials with appropriate properties in the active layer [6], where the mechanisms of photoelectric conversion, absorption, and transport are now well understood [7]. On the other hand, engineering the interface layers requires finding materials able to drive one type of electrical charge toward the electrode while blocking the other [8,9]. Thus the main goals of interfacial layers are the regulation of the electrode work function, the reduction of potential exciton losses, the enhancement of charge carrier extraction [10,11], and the blocking of defect-related recombination events [12,13]. It can also reduce trap formation and smoothen the surface roughness, thus protecting the active layer. Semiconducting metal oxides such as ZnO, TiO_x_, and Cs_2_CO_3_ have been validated as air-stable electron transporting and hole-blocking layers [14,15,16,17]. However, to limit defects with adsorbed oxygen, smoothen the surfaces, improve the device reliability, and reduce the leakage current, conjugated polyelectrolytes, the most widespread being poly (9,9-bis(3-(N,N-dimethylamino)propyl-2,7-fluorene)-alt-2,7-(9,9-dioctylfluorene)) (PFN), are implemented as ETL [18,19,20,21] or casted between the metallic oxide and the active layer [22,23,24]. However, the need for new ETL is still relevant as each BHJ requires interfacial material with highest occupied molecular orbital (HOMO) and lowest unoccupied molecular orbital (LUMO) energy levels, correctly positioned, with respect to the energy level of the *n* type organic semiconductor as acceptor of the BHJ, a still improvable feature. Further developments of tandem devices are of little help since they introduce more complexity in matching energy levels [25,26].

**Figure 1:**
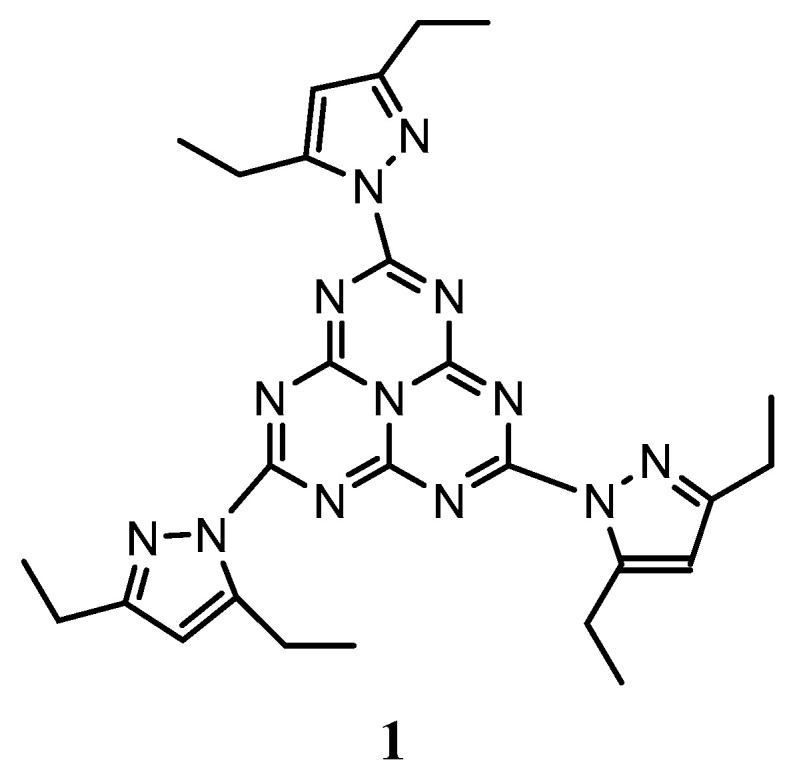
Formula of heptazine 1 used in this work.

Heptazines are electron deficient molecules whose interest is sharply rising, due to their exceptional characteristics like delayed fluorescence [27], photocatalysis [28], and others [29,30,31]. While their syntheses were delicate for a long time, an easy approach to this fascinating family was recently introduced by one of us [32]. In compound 1 (Figure 1), the pyrazole moieties, besides the fact they are exchangeable, are also sufficiently electron-deficient to arouse interest to investigate its use as ETL.

It should be noted that all heptazines substituted with electron-deficient groups are suitable candidates for this general purpose, and this preliminary study is currently extended to other derivatives of this highly promising family. Heptazine-derived materials have already been proposed in OPV devices, but there are in situ formed polymers, with ill-defined structures, while however showing interesting performance [33,34].

In this work we propose a heptazine derivative, referred as compound 1 (Figure 1), with an adapted LUMO level, to be used as interfacial modifier of potential interest in the field of organic electronics. Considering its suitable electronic properties, this molecule could, for example, promote electron injection in classical fullerene-based OPV devices. The synthesis of this heptazine has been recently described by some of us [32]. We show that, beyond energy level matching, this large conjugated molecule with a 3-fold symmetry forms very smooth uniform thin layers of controllable thickness, whether they are processed from physical deposition methods (thermal evaporation under vacuum) or from spin-coating, a rare feature with organic small molecules. The layers have been thoroughly characterized by diverse spectroscopic techniques, including Kelvin probe force microscopy (KPFM) which was used to estimate the influence of a heptazine thin film on the Fermi level (*E_F_*) of a classical transparent electrode substrate (indium tin oxide, ITO). Finally, the potentialities of the heptazine molecule are illustrated by photoluminescence spectroscopy, used to probe the electron transfer efficiency from a reference organic active layer made of tris-(8-hydroxyquinoline) aluminum (Alq3). The results are also discussed in relation to the electrochemical potentials.

It should be also stressed that applications of monomeric heptazines in the field of molecular organics are numerous and not restricted to inverse OPV cells. Concerning the adaptability of Alq3, we have used this compound because it is widely used in organic devices, generally speaking, and fits well with this particular heptazine. As recently demonstrated in our founding article on heptazines [35], the redox potential of this molecular platform can be adjusted through the electron affinity of its substituents. Therefore, it is more than likely that molecular engineering, playing on substitution, will enable adaptation of the new heptazine platform to a vast number of other active layers.

## 2. Results and Discussion

### 2.1. Deposition and Characterization of Heptazine Thin Layers

#### 2.1.1. Optical Characteristics

The heptazines are weakly soluble compounds when they do not possess flexible substituents. However, the heptazine 1 studied in this work is quite soluble in dichloromethane (DCM) thanks to the freely moving aryl substituents. On the other hand, due to its average molecular weight (≈550 g/mol) it can still be sublimated under a relatively low pressure, and at a low enough temperature to prevent degradation. Therefore, we investigated the deposition of these molecules, both by sublimation from powder and by spin-coating from DCM solutions. The layers have been characterized by several types of spectroscopy, in relation to their thickness.

**Figure 2:**
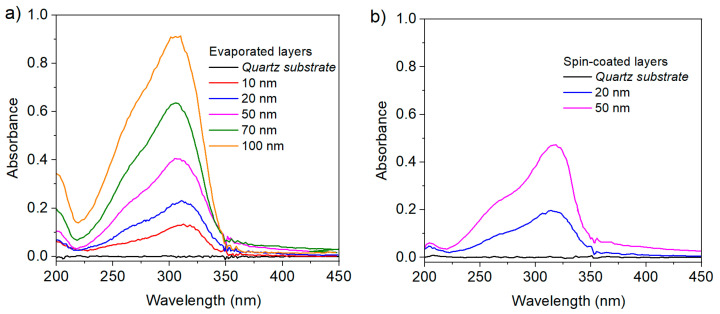
Absorbance of heptazine layers deposited on quartz, in function of the measured thickness. (**a**) Evaporated layers and (**b**) spin-coated layers.

The thickness of the layers can be controlled with reasonable precision, relying on one hand on the deposition time (for the evaporation process), and on the other hand on the spinning rate and the concentration of the solution (for the spin-coating process). Figure 2 represents the absorption of the different heptazine deposited layers, in function of the thickness (measured through a profilometer, see Materials and Methods (Section 3)).

**Figure 3:**
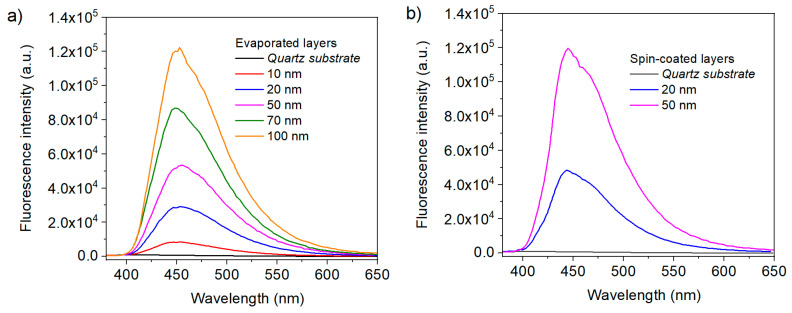
Fluorescence of heptazine layers deposited on quartz, in function of the measured thickness. (**a**) Evaporated layers and (**b**) spin-coated layers.

The fluorescence intensity recovered from the layers (Figure 3) excited at 320 nm, similarly follows the same trend, with quite a remarkable proportionality between the thickness of the layers and the emission.

The quantum yields (QY) for the fluorescence emission have been determined for layers of different thicknesses, and given in Table 1.

They are similar to those measured in solution [32], showing that no aggregation induced quenching occurs with these molecules (as is often the case with high nitrogen content azines). In addition, the QY steadily increases with the film thicknesses, as the probable result of fluorescence quenching for molecules close to the substrate surface; on the other hand, the decrease of luminescence by reabsorption for the thick layers is quite limited, because of the relatively large Stokes shift associated with heptazines. Indeed, the QY for the thickest evaporated layer is exactly the same as previously measured in solution.

The reason for the difference between the emission properties of the spin-coated and evaporated films is not clear at this stage. One possible explanation is probably associated with the inherent different local molecular arrangement in both films, which has also consequences on the global morphology of the deposited films (see next section). The purity of the deposited layer is also quite dependent on the method used, especially comparing a solvent-free deposition method such as vacuum evaporation and spin-coating.

#### 2.1.2. Surface and Morphology Characterizations

**Figure 4:**
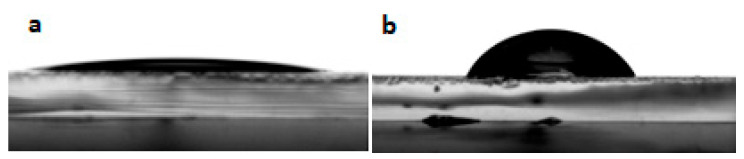
Contact angle on an ITO plate without (**a**) and with (**b**) a deposited heptazine layer.

The wettability of ITO electrodes is a subject of interest for the future realization of organic electronic devices. Organic layers must indeed adhere well to the electrode in order to achieve good electrical contact and limit the delamination of the layers often observed during aging. It is possible to control the wettability of ITO either by physical treatments [36], or by adding self-assembly layers [37,38,39]. The goal is generally to increase the ITO surface energy, and more particularly its polar component. In the context of this work, studying the wettability properties of the modified ITO electrodes is also a relevant tool to assess the presence of heptazine molecules, as they should directly alter the behavior of solvents on the surface. We have performed contact angle measurements to ascertain the modification of the ITO surface by the heptazine thin layer. Figure 4 shows the contact angle of a water drop before (Figure 4a) and after (Figure 4b) heptazine sublimation on glass/ITO, which clearly shows the hydrophobic nature of the surface after modification by the organic heptazine, the contact angle strongly increasing from 20° to 35° ± 1°. This result is consistent with those obtained in the case of conjugated self-assembly layers [37].

**Figure 5:**
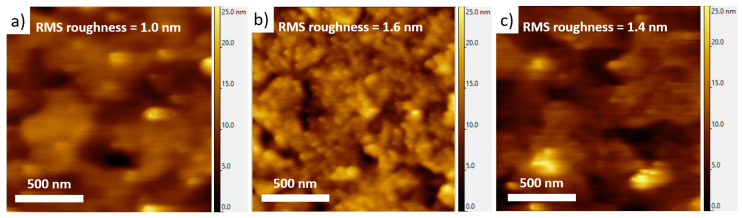
Topographic AFM images of (**a**) bare glass/ITO substrate, and 50 nm thick heptazine layers deposited on glass/ITO by (**b**) vacuum evaporation and (**c**) spin-coating. The corresponding root-mean-square (RMS) roughness values are given in all cases.

The morphology of the films has also been characterized by optical microscopy (OM) and at the nanoscale by atomic force microscopy (AFM). AFM images of both evaporated and spin-coated heptazine films on ITO are given in Figure 5 (the OM images are given as Supplementary Materials, Figure S2).

In both cases, the local morphology is found to be rather smooth on ITO for these 50 nm thick heptazine films, with low root-mean-square (RMS) roughness values below 2 nm. While the homogeneity of the sublimated layer is very good on large scales (see the OM images, Figure S2) as well as at the nanoscale, the spin-coated layer seems to show larger grains or domains, suggesting that the molecular interactions could be slightly different between both techniques. The large-scale morphology of the spin-coated layer, as seen through OM (Figure S2), is also much less homogeneous, which proves that evaporation is a preferred way to prepare even, defect-less thin layers, thus comparable to well-known reference compounds, such as the classical Alq3 metallic complex and a few other organic active molecules. In any cases, the slightly different morphologies induced by the deposition technique are likely to explain the different emission properties of the film revealed in the previous section.

### 2.2. Electronic Properties of the Surface with Heptazine and Ability for Interfacial Charge Transfer

The key parameters for charge injection or extraction when using an interfacial layer to modify a metallic electrode, are the work function of this modified electrode and where the HOMO and LUMO levels of the organic layer referred to as the vacuum level (*VL*) are placed. In this part, we focus on the Fermi level (*E_F_*) determination of an ITO/Heptazine modified electrode by KPFM. As *E_F_* will be situated between the HOMO and the LUMO levels of the organic layer, it will not directly give the extraction potential of the modified electrode since electron transport occurs via the HOMO or the LUMO level of this organic layer. It will just give us insight into whether or not an interfacial dipole layer is present, due to electron exchange between the metal and the organic layer, and if band bending occurs in the depths of the organic layer.

**Figure 6:**
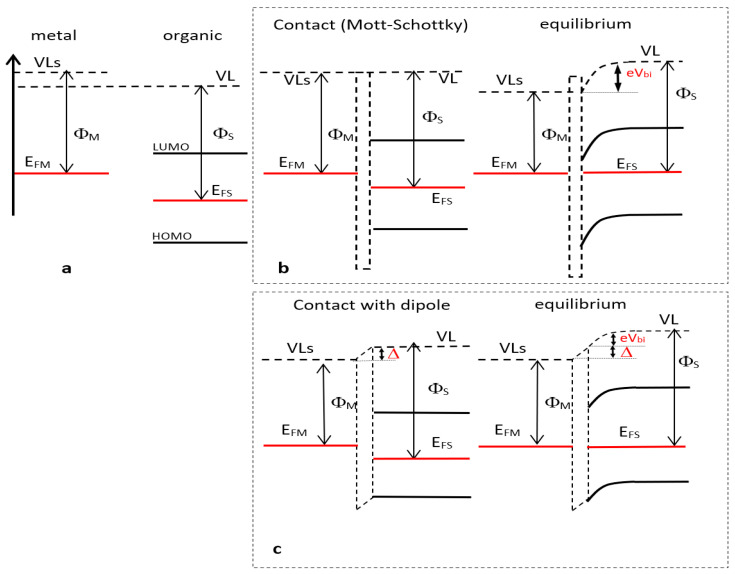
(**a**) Kelvin Probe Force Microscopy (KPFM) measurements of a metal and an organic layer Vacuum Levels (VL’s) for metal are higher than standard VL because of electron environment at surface); (**b**) contact (left) and electronic equilibrium (right) between the electrode and the organic layer according to the Mott–Schottky law; (**c**) contact (left) and electronic equilibrium (right) between the electrode and the organic layer in the case of interfacial dipole.

In the case of a pure metallic electrode, using KPFM we can easily measure the work function $\Phi_{M}$ that corresponds to the difference between the vacuum level at the immediate proximity of the metal surface (noted *VL_S_*) and the metal Fermi level (Figure 6a).

Note that this vacuum level can be different from that of *VL* far from the metal due to the electron-rich environment at the metal surface [40,41,42]. When we add the interfacial layer to the electrode surface, the same KPFM measurement will give Φ*_S_*, the difference between the *VL* and the Fermi level at the surface of the organic layer. In the case of organic layers with poor electron density at the surface, one can assume the vacuum level value will be *VL* (Figure 6a). When the contact is effective between the metal and the organic layer, *VL_organic_* (i.e., *VL*) will take the value of *VL_S_* with (Figure 6b) or without (Figure 6c) continuity between the respective vacuum levels whether or not the contact obeys the Mott–Schottky law. In the case of discontinuity, it is attributed to a spatially-limited electron exchange between the electrode and the organic layer resulting in a dipole moment Δ, the sign of which depends on the dipole orientation. When electrons flow from the electrode to the organic layer (case of Figure 6c, which corresponds to the heptazines layer case) the dipoles are oriented from the organic layer to the metal resulting in a *VL* decay from the organic to the metal and Δ > 0. Moreover, electron exchange will take place between the electrode and the organic layer in order to align the Fermi levels in the two materials at equilibrium provided there are enough charges in the organic part. This condition is generally fulfilled as soon as the organic layer thickness is larger than few nanometers. Consequently, a band bending occurs in the organic part, including *VL*, so that a change in Fermi level values depicted by KPFM between the bare electrode Φ*_M_* and the metal/organic modified electrode Φ*_S_* will denote the presence of a band bending of value *eV_bi_* and possibly an interface dipole Δ (see figure below): $eV_{bi}=\left|\Phi_{M}-\Phi_{S}\right|$ or $eV_{bi}=\left|\Phi_{M}-\Phi_{S}+\Delta \right|$ accordingly [4,43].

We have extensively explored the work function of an ITO electrode modified by thin layers of different thicknesses of heptazine 1, prepared by spin coating or by vacuum sublimation. The Fermi levels are listed in Table 2 below (the measurement made in the case of Au is given for reference).

The measured potentials do not change as could be expected with the layer thicknesses, which is a point in support of the homogeneity of the deposits and reveals that the thicknesses we used are too large to observe any band bending resulting from the electron exchange at the interface. Indeed, band bending does not extend more than a few nanometers in most metal–organic interfaces studied to date [41,42], and its observation requires very sophisticated experiments (UHV in situ measurements), typically out of reach in open air. For the two deposition methods, we clearly see a reproducible shift of the modified electrode work function with values of 0.6 eV and 0.25 eV for evaporated and spin coated layers, respectively. This denotes the presence of a bend bending or an incoming dipole from the organic layer to the metallic electrode due to electron transfer from the metal to the organic and corresponding to a positive value of Δ. This is reasonable taking into account the strong acceptor nature of the heptazine. Note that the presence of a positive dipole at a metal organic interface is particularly uncommon and joins the cases of few very strong organic acceptors like TCNQ or perylene (DP-NTCI) [42]. We also think that the work function shift we obtain is rather due to the cumulative effect of an interface dipole and band bending (Figure 6c) than a band bending alone (pure Mott–Schottky contact, Figure 6b). If it was so, taking into account a 0.6 eV shift and an organic band gap of 3.5 eV (extracted from optical absorption Figure 2) the electrode Fermi level would take place very close to the LUMO of the organics when contact is established, possibly with a pinning of the Fermi level at this LUMO state. In this case, the work function decay would be far higher, towards 1–2 eV. Nevertheless, our potentials seem slightly dependent on the deposition technique. Attempts to relate this to crystallinity failed, since both types of layers did not show any crystallinity, as far as we could investigate by our means (see Supplementary Materials, Figure S2). A more likely interpretation can be proposed considering that interfacial dipole values can be strongly sensitive to any change in the molecular orientation or dielectric permittivity near the interface, which can result from using two different deposition techniques.

### 2.3. Comparative Discussion on the Behavior of Heptazine Layers

With KPFM, the work function of surfaces can be observed at a scale of a few nanometers. The difference in Fermi levels between the AFM tip and the sample surface leads to a current flowing until Fermi levels are aligned. Applying a voltage between the tip and the sample to compensate for this contact difference potential (CDP) allows extraction of the Fermi level of the sample, knowing that of the tip (which can be derived from a suitable calibration using reference samples). The notion of Fermi level in a molecular material is more elusive, but it can be considered that it lies in the middle of the gap for undoped materials, so that $E_{F}$ is equal to $\left(E_{LUMO}-E_{HOMO}\right)/2$.

This value can be correlated to the results already obtained in fluorescence and electrochemistry. We can identify the $E_{LUMO}$ to the reduction redox potential, applying the correspondence formula $E_{orbital}=-\left(-4.55+V_{redox}\right)$, $V_{redox}$ being the electrochemical potential in solution [42,43], and $E_{orbital}$ measured with origin at vacuum level, the classically admitted 4.55 V representing the difference between the vacuum level and the saturated calomel electrode (SCE) [44], (4.3 V being the difference between the normal hydrogen electrode (NHE) and vacuum, and 0.25 V the additional difference between the NHE and the SCE). Of course, these comparisons stand within the formal errors due with the fact that solvent effects are neglected, but are usually reasonably meaningful in the case of conjugated aromatic molecules, where the solvation effects are limited. The fluorescence measurements give the HOMO–LUMO gap, from the intersection of both absorption and emission spectra, from which the HOMO energy is in turn easily calculated. From our previous work we know that the electrochemical potential of 1 is equal to −1.23 V, and its optical gap is 3.5 eV; we thus obtain $E_{LUMO}=-3.32\;\mathrm{eV}$ and $E_{HOMO}=-6.82\;\mathrm{eV}$. The Fermi level can therefore be calculated of 5.02 eV. Taking into account the vacuum level shift (0.6 eV in the case of spin coated layers, 0.25 eV in the case of evaporated layers), the value of which having to be added to $\Phi_{S}$ (Figure 6c), we obtained heptazine Fermi levels with the values of 5.67 eV and 5.32 eV, respectively, if the layer was intrinsic (i.e., $E_{FS}=\left(E_{LUMO}-E_{HOMO}\right)/2$). The measured values are inferior of 0.1–0.2 eV, which denotes a slightly higher Fermi level, corresponding to an n doping of the layer on ITO. Indeed, this approach appears quite reasonable taking into account the strong acceptor nature of the heptazine.

While it can be considered that the evaporated layers form a purer material, probably even more organized after the low temperature annealing step, those values can be considered as remarkably concordant for evaluating the absolute position of frontier orbitals in the solid-state compared to solution, in the case of such molecular materials thin layers.

### 2.4. Influence of Heptazine Thin Layers on Electron Transfer Rates from An Alq3 Thin Films

Of course, a step beyond the in-depth evaluation of the heptazine layer’s optical and electrical behavior, was to investigate their behavior in the presence of an active organic layer, such as an OPV blend or an emissive organic layer in the case of light-emitting diodes for example. In this direction, Alq3 layers were a reasonable first choice as a proof of concept, since they show compatible energy levels and since they can be easily deposited on top of the heptazine layers using thermal evaporation. Moreover, Alq3 layers absorb visible light where heptazines do not, allowing a simple characterization of the charge transfer mechanisms by photoluminescence spectroscopy [45,46,47].

**Figure 7:**
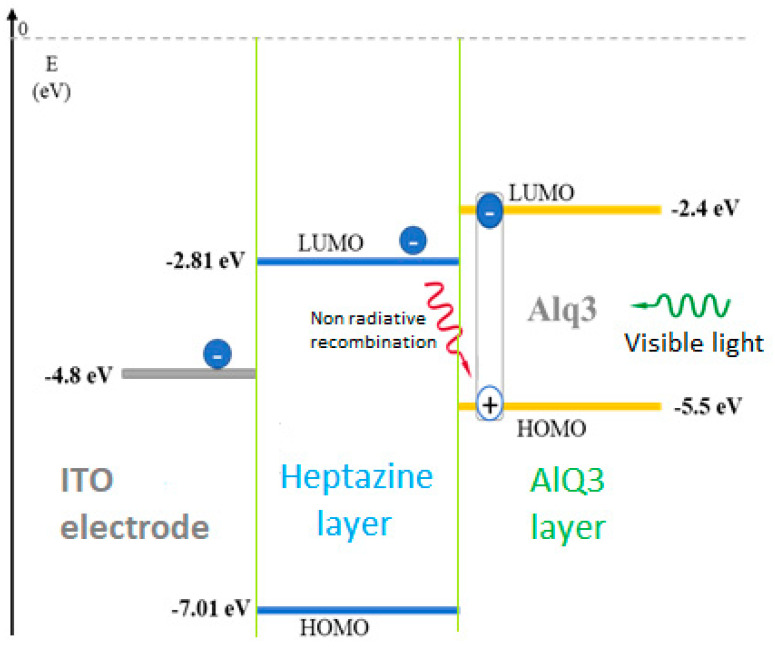
Scheme of the electron transfer from an activated Alq3 layer into an underlying sandwiched accepting heptazine 1 layer, deposited on an ITO electrode.

An efficient means of investigation is to monitor the emission of the Alq3 layer under visible excitation at 404 nm, whether it is deposited on an ITO or an ITO/heptazine electrode. Indeed, the emission properties of Alq3 on specific substrates can reveal the charge transfer mechanisms occurring at the interface, which are responsible for a significant PL extinction. Such charge transfer processes are preliminary steps required for efficient charge extraction in a final device, assuming a reasonable charge mobility (which is currently being assessed, while being out of the scope of the present study). To illustrate our strategy, Figure 7 below represents the proposed energy configuration of the materials, as well as the expected photo-induced charge transfers mechanism from Alq3.

Charges extracted from the Alq3 layer at the interface can be extracted in the heptazine layer and later on, either driven to the ITO electrode, or lost by non-radiative recombination with the oxidized Alq3 layer, therefore decreasing its PL emission. We note that it was important to reduce the thickness of the Alq3 layer, considering the exciton diffusion length of the material usually reported (between 3 and 25 nm) [48].

**Figure 8:**
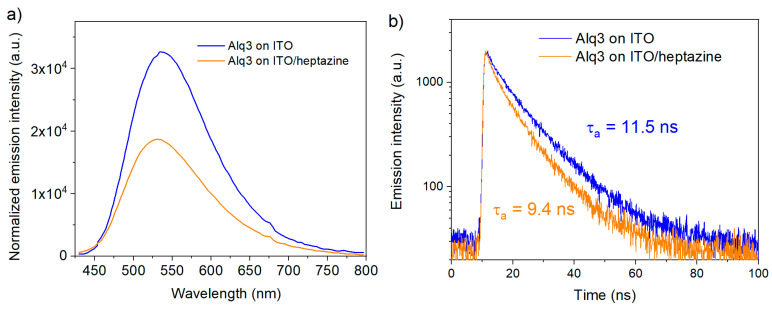
Photoluminescence emission (**a**) and TRPL (time-resolved photoluminescence) decay curves (**b**) of 10 nm thick Alq3 layer deposited on ITO and on an ITO/heptazine (50 nm) substrate.

Figure 8a below represents the change in the emission of the Alq3 layer deposited on top of a heptazine layer in the case of an ITO substrate, using a 404 nm excitation (the case of a glass substrate is given as Supplementary Materials, Figure S3a).

In both cases, the emission of the Alq3 layers are significantly reduced when the fluorophore is deposited onto the heptazine layer, in comparison to the case where it is deposited directly either on glass (Figure S3a) or ITO (Figure 8a). This trend is confirmed by transient photoluminescence measurements made on the same samples, which show a significantly reduced Alq3 exciton lifetime in the presence of heptazine (average lifetime of 11.5 ns without heptazine versus 9.5 ns with heptazine, see Figure 8b for samples deposited on glass/ITO substrates, and Figure S3b in ESI for samples deposited on glass substrates).

Both results demonstrate that fluorescence quenching is much more pronounced in the presence of the heptazine interfacial layer. Actually, since the excitation takes place at 404 nm, a wavelength where the heptazine is not absorbing, the quenching process mainly occurs by photo-induced charge transfer to the heptazine layer. Both experiments on glass and on ITO (see Supplementary Materials, Figure S3) provide quite similar features.

This fact that similar observations are made for ITO and bare glass substrates indicates that charge extraction down to ITO is a very minor process compared to non-radiative recombination at the Alq3 interface, immediately after photo-excitation. The relatively thick layer of heptazine layer (50 nm) is certainly a drawback for efficient charge collection by the electrode (Figure 7). This assumption is also confirmed through thickness-dependent PL and time-resolved photoluminescence (TRPL) measurements made on samples of varying heptazine layer thicknesses (from 10 to 100 nm; see Supplementary Materials, Figure S4). Similar Alq3 emission properties (emission intensity and TRPL decay kinetics) are observed for thin and thick heptazine interfacial layers. It indicates relatively limited charge mobility in the heptazine layer, although a specific characterization is required on this aspect.

## 3. Materials and Methods

### 3.1. Synthesis of Heptazine Molecules

Heptazine 1 preparation has been reported recently by one of us [32].

### 3.2. Elaboration and Characterization of Heptazine Thin Films

The heptazine 1 studied in this work is quite soluble in dichloromethane DCM (Carlo Erba, Spain). On the other hand, due to its average molecular weight (≈500 g/mol), it can be sublimated under a relatively low pressure, at a temperature where the molecules are still stable. Therefore, we investigated the deposition of these molecules, both by sublimation under vacuum and by spin-coating from DCM solutions. The solutions were prepared following these steps: 15 mg of heptazine (weighed with a microbalance) were solubilized into 3 mL of DCM in a tight vial. After quick hand mixing, the vial was shaken first on a mechanic stirrer, then sonicated for at least 15 min in a standard water sonicating bath.

To prepare heptazine films by sublimation, the process described below was followed. The heptazine was placed in a dedicated tungsten crucible, in an Edwards Auto 306 high vacuum evaporator. The pressure was lowered to about 10^−6^ mbar, then the current (provided by a current source) was gradually increased up to 8 A, following two 4 A steps of 5 min each. When 8 A was reached, the current was increased by smaller steps of 1 A every 3 min. At 12 A, the deposition speed reached an approximately stable rate of 0.1 nm/s and the conditions were kept constant until obtention of the desired thickness, ranging from 10 to 100 nm (accuracy of ±5 nm), as measured by a mechanical profilometer (DEKTAK XT, Bruker, Switzerland).

### 3.3. Spectroscopic Characterizations

#### 3.3.1. UV-Visible Absorption Spectroscopy and Optical Microscopy Images

UV-visible absorption spectra of heptazine thin layers were recorded in transmission mode using an AGILENT Cary 300, (Santa Clara, CA, USA) spectrometer equipped with an integrating sphere. The baseline was defined by the bare substrate priori to thin film measurements. Optical images of thin films on various substrates were recorded using a LEICA DM12000 M microscope, (Dresden, Germany)

#### 3.3.2. Photoluminescence Studies

Photoluminescence (PL) spectroscopy measurements on solutions and solid-state films were performed using an FLS980 spectrometer from Edinburgh Instruments (Edimburgh, UK). Steady-state PL was performed using a monochromated 450 W Xenon lamp and a cooled Hamamatsu R928P photodetector. Samples were placed at 45° in a specific sample holder, ensuring similar excitation conditions for all heptazine films (λ_exc_ = 320 nm). The spectral resolution of the emission monochromator was set to 2 nm and the excitation intensity was chosen so that no damages were observed on the samples over time. Absolute PL emission quantum yields (PLQY) of both solutions and thin films deposited on quartz substrates were estimated using an integrating sphere. Charge injection rates on heptazine/Alq3 samples were estimated using Time-Resolved Photoluminescence (TRPL) spectra recorded on the same apparatus using a 404 nm picosecond laser diode (temporal width of 150 ps) and by detecting the emission at 533 nm (2.33 eV) using Time-Correlated Single Photon Counting (TCSPC). PL decays were adjusted using bi-exponential decay functions, which were subsequently used to evaluate an average decay time *τ_a_* [49]. The instrument response function (full width at half maximum of ~520 ps), measured using a diffusive reference sample LUDOX HS-40, (Sigma Aldrich, Buchs, Switzerland) was systematically deconvoluted for each TRPL spectrum.

#### 3.3.3. AFM and KPFM Analysis

The nanoscale morphology and work function of the heptazine thin films were recorded using a Nano-Observer atomic force microscope (AFM) from CSI Instruments. While topographic images were recorded in tapping mode, the Fermi level of heptazine layers deposited on glass/ITO substrates was performed using Kelvin probe force microscopy (KPFM) measurements. Platinum-coated AFM tips with a resonant frequency between 43 and 81 kHz and elastic coefficient of 1.5 N/m were used to determine the contact potential difference *V_CPD_* between the tip and the ITO/heptazine sample. Measurement was performed in amplitude modulation mode and single pass KPFM mode in ambient conditions; thereby *V_CPD_* between the tip and the sample was mapped simultaneously to the topography (*V_CPD_* is measured as the maximum potential change of the tip required to null the alternating current, between tip and heptazine, resulting from the oscillating capacitance). *V_CPD_* is defined as: $V_{CPD}=\frac{\phi_{tip}-\phi_{sample}}{-e}$ where $\phi_{tip}$ and $\phi_{sample}$ are the work functions of the tip and the sample, respectively; and *e* the elementary charge. As a calibration is needed for absolute measurements by KPFM, we measured the contact potential difference between the tip and a fresh gold layer evaporated on glass, as well as with a highly oriented pyrolytic graphite (HOPG) plate. Assuming that $\phi_{Au}=5.1\;\mathrm{eV}$ and $\phi_{HOPG}=4.47\;\mathrm{eV}$ [50], the tip work function was calculated for each AFM tip used in this work.

## 4. Conclusions

This study describes, for the first time, the deposition of molecular heptazine thin layers, as well as their physical properties, based on fluorescence measurements and Fermi level assessment by Kelvin probe force microscopy. A relevant estimation of their energetic configuration as thin film has been obtained, which allows us to propose their use as interfacial layers in organic devices such as solar cells. As a proof of concept, we built a simple model device where heptazine thin films are promoting charge transfer process from an Alq3 photo-active layer, as revealed by steady-state and transient photoluminescence spectroscopy. Although only one heptazine molecule has yet been studied in this respect, the results gathered are already encouraging, since they clearly suggest the possibility of using this new family of molecules as interfacial modifiers in organic optoelectronic devices. In particular, it is likely that substituents engineering could still noticeably improved the efficiency of this new family in the field of molecular electronics. New molecules of this emerging family are currently under investigation.

## Figures and Tables

**Table 1:** QY of the deposited layers as a function of layer thicknesses.

Deposition Technique	Thickness	Photoluminescence Quantum Yield (QY)
Evaporation	20 nm	3.5%
Evaporation	80 nm	8.3%
Evaporation	100 nm	9.2%
Spin Coating	25 nm	9.3%
Spin Coating	50 nm	13.8%
Spin Coating	70 nm	14.2%
Spin Coating	100 nm	15%

**Table 2:** Extraction potential values for the different heptazine layers, including the gold and ITO references. Top, evaporated layers; bottom, spin-coated layers.

Sample	Thickness	Fermi Level
Au	_	5.1 eV
ITO	_	4.86 eV ± 50 meV
ITO/Heptazine	10 ± 5 nm	5.49 eV ± 35 meV
ITO/Heptazine	20 ± 5 nm	5.5 eV ± 35 meV
ITO/Heptazine	46 ± 5 nm	5.45 eV ± 40 meV
ITO/Heptazine	70 ± 5 nm	5.45 eV ± 40 meV
Au	_	5.1 eV
ITO	_	4.89 eV ± 30 meV
ITO/Heptazine	25 ± 10 nm	5.2 eV ± 20 meV
ITO/Heptazine	50 ± 10 nm	5.12 eV ± 20 meV
ITO/Heptazine	100 ± 10 nm	5.09 eV ± 20 meV

## Supplementary Materials

The following are available online at https://www.mdpi.com/1996-1944/13/17/3826/s1, Figure S1: Comparison by optical microscopy of different types of heptazine layers, according to the deposition conditions. The film thickness is around 50 nm in all cases. (a) ITO neat substrate, (b) Spin-coated heptazine on ITO, (c) Same as (b), but with 20 min annealing at 90° (d) Heptazine spin coated on a heated (90 °C) substrate, then annealing at 90 °C, (e) Heptazine evaporated on ITO (see Materials and Methods). Scale bar: 0.5 mm, Figure S2: XRD diagram of heptazine 1 powder and thin films. Note: the black and blue traces are overlapping, Figure S3: PL emission (a) and TRPL decay curves (b) of Alq3 deposited on glass or on glass/heptazine substrate, Figure S4: PL emission (a) and TRPL decay curves (b) of Alq3 deposited on ITO/heptazine substrates as a function of the heptazine layer thickness. The excitation is made at 404 nm in both cases, Table S1: Optimized deposition parameters for the realization of heptazine thin films by spin coating, Table S2: Quantitative analysis of TRPL decay curves of Alq3 emission deposited on glass/ITO or on glass/ITO/heptazine substrate.
