# Supplementary material for: Characterization and Electronic Properties of Heptazine Layers: Towards Promising Interfacial Materials for Organic Optoelectronics
Source: Materials (Basel). 2020 Aug 29;13(17):3826. doi: 10.3390/ma13173826 (PMC7504471; doi:10.3390/ma13173826)
Supplement: Supplementary file 1 [file materials-13-03826-s001.pdf]

materials

Supplementary Information

Characterization and Electronic Properties of Heptazine Layers: Towards Promising Interfacial Materials for Organic Optoelectronics

1. Deposition of Heptazine Thin Films by Spin-Coating

Heptazine thin films were prepared by spin-coating from solution or by thermal sublimation under vacuum (see the Materials and Methods section in the main article). For the deposition from solution, some optimizations were conducted in order to achieve homogeneous films with suitable thicknesses, both on pre-cleaned glass/ITO and quartz substrates. Table S1 below presents the corresponding parameters, including post-annealing conditions and achieved layer thickness, as extracted from mechanical profilometry measurements (DEKTAK XT, Bruker, accuracy ± 5 nm).

Table S1. Optimized deposition parameters for the realization of heptazine thin films by spin coating.

Substrate	Speed (RPM)	Acceleration (RPM/s)	Time (s)	Annealing Time 90 °C (min)	Solution Concentration (mg/mL)	Film Thickness (nm)
Glass/ITO	2000	1000	50	20	2	20
Glass/ITO	4000	1000	50	20	5	50
Quartz	2000	1000	50	20	2	20
Quartz	4000	1000	50	20	5	50

2. Optical Microscope (OM) Images of Heptazine Thin Films

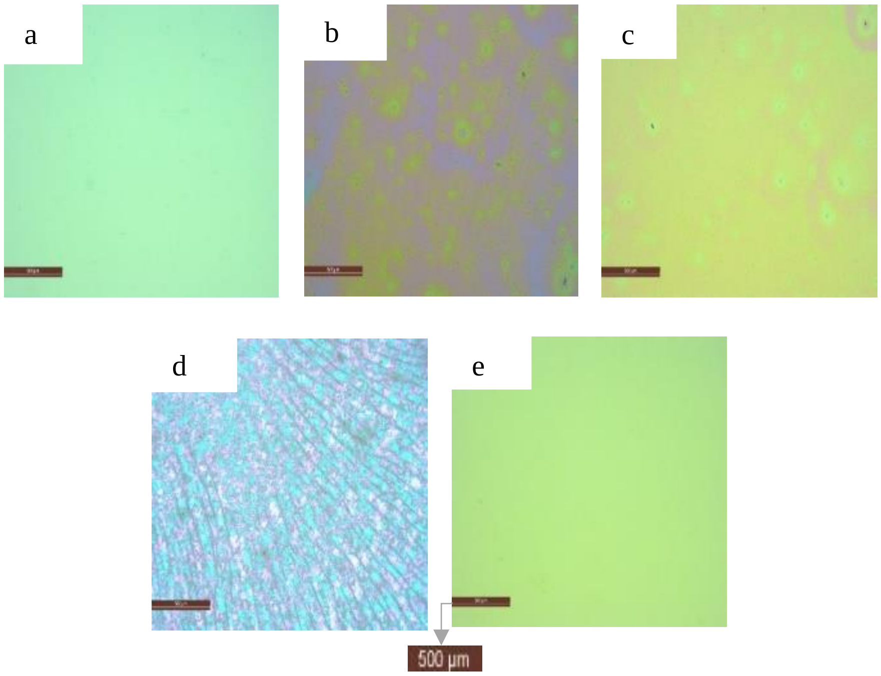

Figure S1. Comparison by optical microscopy of different types of heptazine layers, according to the deposition conditions. The film thickness is around 50 nm in all cases. (**a**) ITO neat substrate, (**b**) Spin-coated heptazine on ITO, (**c**) Same as (**b**), but with 20 min annealing at 90° (**d**) Heptazine spin coated on a heated (90 °C) substrate, then annealing at 90 °C, (**e**) Heptazine evaporated on ITO (see Materials and Methods). Scale bar: 0.5 mm.

3. Crystallographic Data of Heptazine Thin Films Deposited on Glass Substrates

X-ray diffraction patterns (XRD) of heptazine films deposited on glass substrates were recorded using the D-8 Advance Bruker diffractometer (Karlsruhe, Germany). The measurement was made in ambient conditions, a few hours after thin film deposition. The XRD diffractogram corresponding to heptazine powder is also given for reference (Figure S2).

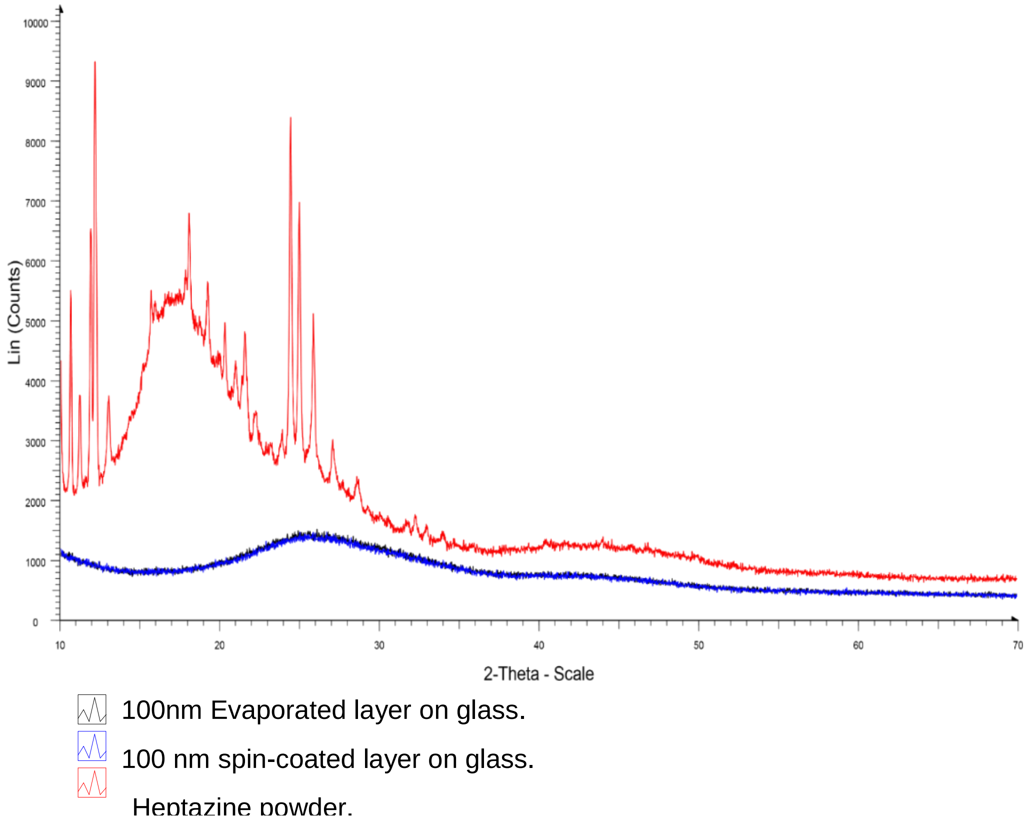

Figure S2. XRD diagram of heptazine 1 powder and thin films. Note: the black and blue traces are overlapping.

4. Time-Resolved Photoluminescence (TRPL) Measurements on Glass Substrates

TRPL measurements were performed on both glass/ITO and glass substrates (see the Materials and Methods section of the main article). Figure S3 below presents the PL emission and TRPL spectra of Alq3 deposited on glass and glass/heptazine substrates.

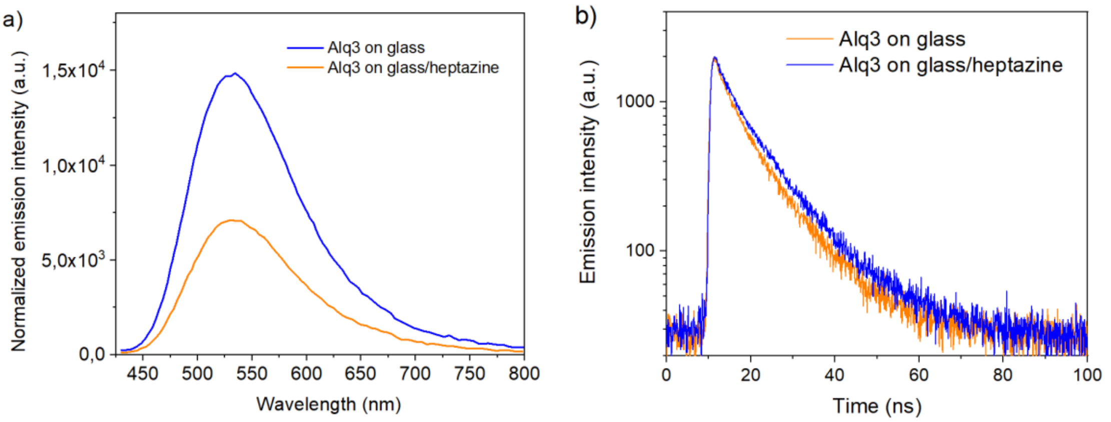

Figure S3. PL emission (**a**) and TRPL decay curves (**b**) of Alq3 deposited on glass or on glass/heptazine substrate.

Measurements made on glass/ITO substrates are presented in the main article, in Figure 8. In this case, a simple quantitative analysis of the TRPL decays was conducted based on the charge transfer efficiency defined below, where τ_a is the average decay time [1,2]:

$$\eta_{TE} = 1 - \frac{\tau_a(Heptazine,\ Alq3)}{\tau_a(Alq3)} \quad \text{(S1)}$$

Table S2. Quantitative analysis of TRPL decay curves of Alq3 emission deposited on glass/ITO or on glass/ITO/heptazine substrate.

Substrat	$\tau_a(Alq3)$	$\tau_a(Heptazine, Alq3)$	η_{TE}
Verre/ITO	11.5 ns	9.4 ns	18.6%

The charge transfer efficiency is therefore improved by a factor of nearly 20% in the presence of heptazine, compared to a reference sample based on ITO/Alq3.

Finally, Figure S3 below presents the PL emission and TRPL decay curves of an Alq3 layer deposited on ITO/heptazine substrate, as a function of heptazine layer thickness. Apart from small sample-to-sample variations of the absolute emission intensity (no integrating sphere was used in this case), rather similar emission properties (intensities and lifetime) are evidenced in all cases, indicating that the exciton lifetime in Alq3 in mainly governed by the heptazine/Alq3 interface.

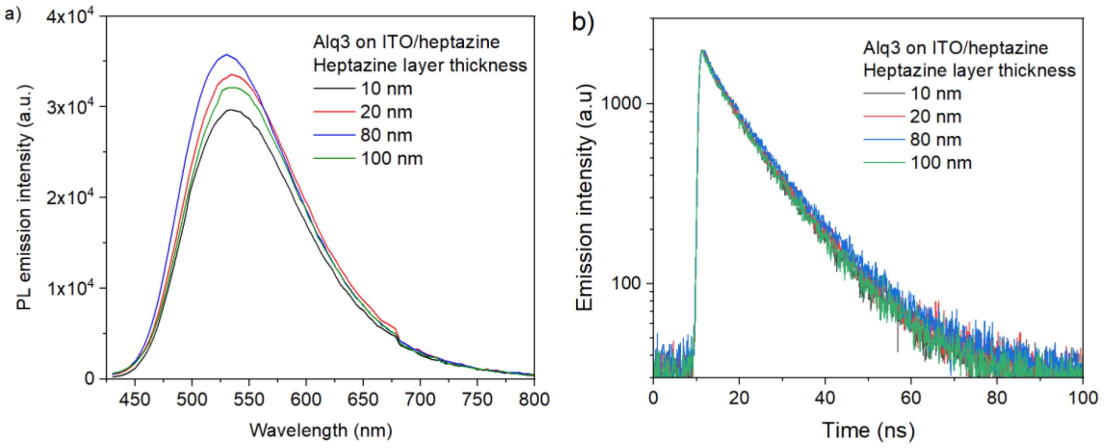

Figure S4. PL emission (**a**) and TRPL decay curves (**b**) of Alq3 deposited on ITO/heptazine substrates as a function of the heptazine layer thickness. The excitation is made at 404 nm in both cases.

References

1. Aldakov, D.; Sajjad, M.T.; Ivanova, V.; Bansal, A.K.; Park, J.; Reiss, P.; Samuel, I.D.W. Mercaptophosphonic acids as efficient linkers in quantum dot sensitized solar cells. *J. Mater. Chem. A* **2015**, *3*, 19050–19060, doi:10.1039/C5TA04021C.
2. Pham, T.T.T.; Saha, S.K.; Provost, D.; Farré, Y.; Raissi, M.; Pellegrin, Y.; Blart, E.; Vedraine, S.; Ratier, B.; Aldakov, D.; et al. Toward Efficient Solid-State p-Type Dye-Sensitized Solar Cells: The Dye Matters. *J. Phys. Chem. C* **2017**, *121*, 129–139, doi:10.1021/acs.jpcc.6b10513.
